# Chronic Kidney Disease on Health-Related Quality of Life in Patients with Diabetes Mellitus: A National Representative Study

**DOI:** 10.3390/jcm10204639

**Published:** 2021-10-10

**Authors:** Yun Soo Hong, Hoon Kim, Di Zhao, Ajin Cho

**Affiliations:** 1 Departments of Epidemiology and Medicine, and Welch Center for Prevention, Epidemiology, and Clinical Research, Johns Hopkins University Bloomberg School of Public Health, Baltimore, MD 21205, USA; hong.yunsoo@jhu.edu (Y.S.H.); dizhao@jhu.edu (D.Z.); 2 Department of Obstetrics and Gynecology, Seoul National University College of Medicine, Seoul 03080, Korea; obgyhoon@gmail.com; 3 Department of Obstetrics and Gynecology, Seoul National University Hospital, Seoul 03080, Korea; 4 Department of Internal Medicine, Division of Nephrology, Hallym University College of Medicine, Kangnam Sacred Heart Hospital, Seoul 07441, Korea

**Keywords:** diabetes mellitus, chronic kidney disease, health-related quality of life, EQ-5D

## Abstract

Importance: With an increasing prevalence of diabetes mellitus (DM) and comorbid chronic kidney disease (CKD), health-related quality of life (HRQoL) in patients with DM and CKD needs to be better understood. Objective: To investigate the association between the severity of CKD on HRQoL in DM patients. Design: A cross-sectional study of a nationally representative population-based survey, the Korea National Health and Nutrition Examination Survey (KNHANES). Setting: Data collected between 2007 and 2018 from the KNHANES. Participants: Adult participants with DM who completed the self-administered European Quality of Life Questionnaire Five Dimension (EQ-5D) questionnaire (*n* = 7243). Exposures: CKD stages defined by the Kidney Disease Improving Global System (KDIGO) staging system. Main Outcomes and Measures: We estimated the odds ratios (ORs) and 95% confidence intervals (CIs) of the presence of having problems in the 5 dimensions (mobility, self-care, usual activities, pain/discomfort, and anxiety/depression) of EQ-5D by CKD stage after adjusting for socio-demographic parameters and comorbid conditions. In addition, the EQ-5D index, reflecting the overall health status, was compared across CKD stages. Results: Among 7243 participants (mean (standard error) age 58.2 (0.2) 56.9% male), 24.0% (*n* = 1768) had CKD and 8.6% (*n* = 775) had stage 3–5 CKD. Pain/discomfort was the most common problem (30.5%) among patients with DM. Participants with more advanced CKD were more likely to experience problems in all dimensions of EQ-5D except the anxiety/depression dimension. In particular, compared to those without CKD, the adjusted ORs (95% CI) for any problem in the usual activities dimension was 1.65 (1.30, 2.10) in CKD stage 3 and 4.23 (2.07, 8.67) in CKD stage 4–5. Moreover, participants with stage 3 (−0.016 (−0.029, −0.003)) and stage 4–5 CKD (−0.088 (−0.129, −0.048)) had significantly lower EQ-5D index than those without CKD. However, compared with no CKD, CKD stage 1–2 was not significantly associated with having any problem in any dimensions. Conclusions and Relevance: In this nationally representative study, patients with DM had a high prevalence of self-reported poor HRQoL and the prevalence increased with more advanced stages of CKD. Therefore, assessment of HRQoL and interventions are necessary at early stages of CKD in DM patients.

## 1. Introduction

Diabetes Mellitus (DM) is a global epidemic and an increasing public health concern. DM is the eighth leading cause of death and is estimated to affect about 460 million people globally [1]. The global prevalence of DM in adults is also estimated to increase from 8.8% in 2017 to 9.9% by 2045 [2]. Furthermore, DM is a chronic and progressive condition that leads to multiple complications, such as cardiovascular disease (CVD), chronic kidney disease (CKD), retinopathy, and neuropathy [3,4]. Patients with DM, therefore, have an increased burden due to its negative effects on the aspects of physical, psychological, and social well-being [5].

CKD is a common complication of DM, affecting about 25–40% of patients [6,7,8]. The presence of CKD in patients with DM further increases the risk of CVD, progression to end-stage renal disease (ESRD), and mortality [9,10]. Patients with DM-CKD also experience a wide range of physical and psychological symptoms that may have serious adverse effects on their quality of life [11]. For instance, the majority of CKD patients experience bone and joint pain, muscle cramps, sadness, irritability, and difficulty sleeping. 

Health-related quality of life (HRQoL) is an indicator of an individual’s perceived status of life and well-being. Among patients with DM, complex treatment regimens and strict glycemic control as well as reduced physical and social activities due to diabetic complications have a significant impact on the HRQoL [12,13,14,15]. Despite the high prevalence of CKD in patients with DM and its debilitating nature, little is known about the impact of CKD on HRQoL in this population. 

Measurement of HRQoL using the European Quality of Life Questionnaire Five Dimension (EQ-5D) provides a comprehensive and quantitative evaluation of the patient’s health status in physical and emotional dimensions beyond laboratory measurement [16]. Evaluation of HRQoL among DM patients with CKD will identify their disease burden and allow clinicians to seek proper management to improve their HRQoL. We therefore aimed to investigate the association between the severity of CKD on HRQoL in patients with DM using a nationally representative population-based survey.

## 2. Methods

### 2.1. Study Population

**Figure 1:**
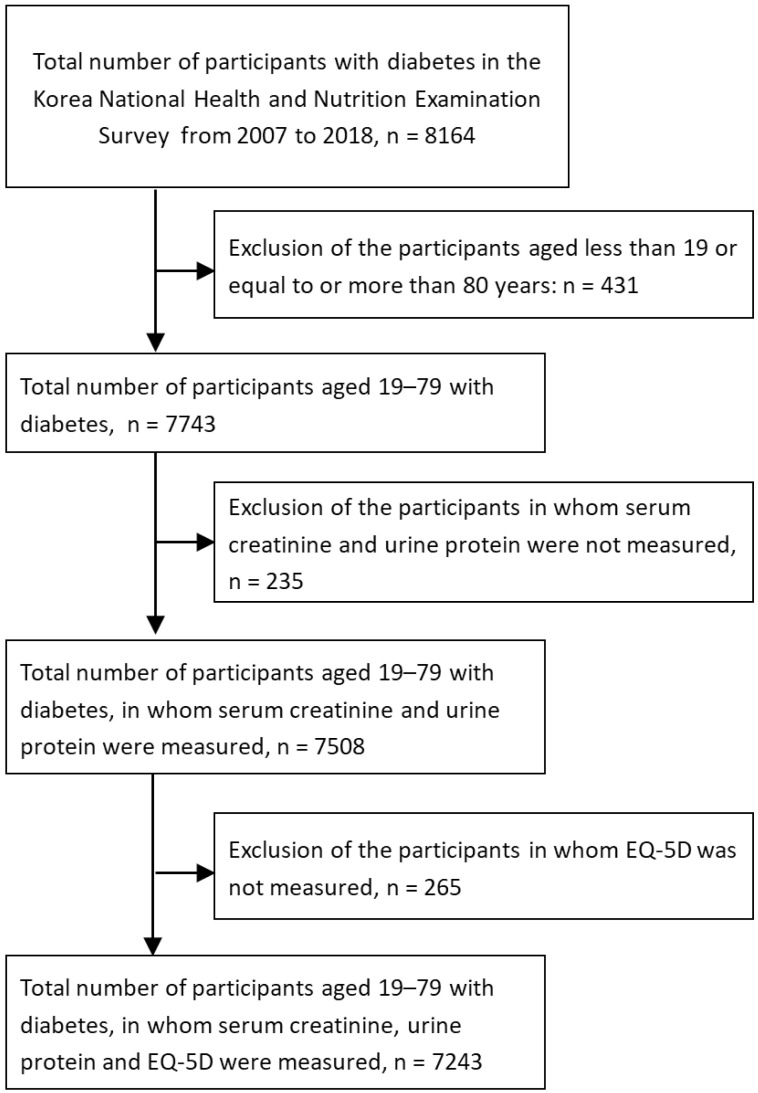
Flowchart of study participants.

The Korea National Health and Nutrition Examination Survey (KNHANES) is an annual nationwide cross-sectional survey designed to investigate the health and nutritional status of the general, non-institutionalized South Korean population. Household interviews and health examinations are performed by trained staff using standardized protocols. We used data from continuous KNHANES between 2007 and 2018. Of the 8164 participants with DM during this period, we excluded participants according to the following criteria: <19 years or ≥80 years (*n* = 431); participants without serum creatinine or urine protein measurement (*n* = 235); and participants who did not complete the EQ-5D questionnaire (*n* = 265). Finally, a total of 7243 adults with DM were included in the present study (Figure 1). 

The institutional review board (IRB) at the Korea Center for Disease Control and Prevention approved the fourth to seventh cycles of KNHANES, 2007–2018 (Approval No. 2007-02CON-04-P, 2008-04EXP-01-C, 2009-01CON-03-2C, 2010-02CON-21-C, 2011-02CON-06-C, 2012-01EXP-01-2C, 2013-07CON-03-4C, 2013-12EXP-03-5C and 2018-01-03-P-A). All study participants submitted an informed consent before participating in the KNHANES, and all procedures were conducted according to the ethical principles of the Declaration of Helsinki. The IRB of Kangnam Sacred Heart Hospital also approved this study (IRB No: 2020-12-006).

### 2.2. Anthropometric and Laboratory Data

After 5 min of rest in a seated position, blood pressure was measured manually 3 times with 30 s intervals. Body mass index (BMI) was calculated by weight in kilograms divided by height in meters squared. 

Blood samples were collected after fasting for at least 8 h and random spot urine samples were collected on the same visit. After being properly processed and refrigerated, they were transported in cold storage to the central laboratory within 24 h of collection. Glucose levels were measured using the hexokinase ultraviolet method with a Hitachi Automatic Analyzer 7600-210 (Hitachi, Marunouchi, Japan). Hemoglobin A1c (HbA1c) levels were measured with a high-performance liquid chromatography assay with a Tosoh G8 instrument (Tosoh, Shiba, Japan) [17]. Serum creatinine levels were measured by the colorimetric method using ADIVIA 1650 (Siemens, Malvern, PA, USA) in 2007 and the Jaffe rate-blanked and compensated method using the Hitachi Automatic Analyzer 7600 (Hitachi, Tokyo, Japan) in 2008–2018. Serum creatinine concentrations were calibrated to isotope-dilution mass spectrometry-traceable standard.

### 2.3. Definition of DM and Kidney Function

We defined DM as the presence of self-reported physician diagnosis of diabetes, self-reported use of any oral hypoglycemic agent or insulin, or having fasting serum glucose ≥126 mg/dL or HbA1c ≥6.5% at the survey visit. We used the Chronic Kidney Disease-Epidemiology Collaboration (CKD-EPI) equation from calibrated serum creatinine measurements to calculate the estimated glomerular filtration rate (eGFR) [18]. Proteinuria was defined as ≥1+ on a dipstick testing. Based on the eGFR and the presence of proteinuria, we classified participants into five stages according to the Kidney Disease Improving Global System (KDIGO) staging system [19]. CKD stage 1 was defined as an eGFR ≥90 mL/min/1.73 m^2^ with proteinuria. CKD stage 2 was defined as an eGFR between 60 and 89 mL/min/1.73 m^2^ with proteinuria. CKD stages 3a (45–59 mL/min/1.73 m^2^), 3b (30–44 mL/min/1.73 m^2^), 4 (15–29 mL/min/1.73 m^2^) and 5 (less than 15 mL/min/1.73 m^2^) were categorized based on eGFR, regardless of the presence of proteinuria. CKD stages 3–5 were further combined and classified as moderate to severe CKD in this study. 

### 2.4. Demographic and Clinical Characteristics

Demographic and clinical characteristics of the participants were collected through a questionnaire. Residential area was defined as urban or rural based on the administrative district [20]. Education level was categorized as elementary, middle school, high school, or college graduates. Annual household income was categorized into quintiles. Smoking status was categorized as never smoker, ex-smoker, or current smoker. High-risk alcohol drinking was defined as >2 times/week and ≥7 drinks at a time for men and >2 times/week and ≥5 drinks at a time for women. Physical activity was calculated using the total metabolic equivalent (MET) based on the International Physical Activity Questionnaire-short form (IPAQ-SF) and categorized as low (<3 METs) or moderate (3–5.9 METs) to vigorous (≥6 METs) physical activity [21].

Hypertension was defined as a self-reported physician’s diagnosis, a self-reported use of anti-hypertensive medication, or systolic blood pressure ≥140 mmHg or diastolic blood pressure ≥90 mmHg on their physical examination. The presence of stroke, coronary heart disease (CHD), history of cancer, asthma, chronic obstructive pulmonary disease (COPD), and arthritis was based on self-report. Non-alcoholic fatty liver disease (NAFLD) was defined using hepatic steatosis index (HSI), which is a noninvasive screening tool for NAFLD with sensitivity and specificity of 93.1% and 92.4%, respectively [22,23,24]. HSI is calculated as 8 × (alanine aminotransferase/aspartate aminotransferase ratio) + BMI (+2 for females, +2 for diabetes patients). NAFLD was defined as HSI > 36.

### 2.5. HRQoL Measurement 

HRQoL was evaluated using EQ-5D [16]. EQ-5D is a widely used self-administered instrument to measure HRQoL and has been validated in many countries including Korea. It consists of five dimensions: mobility, self-care, usual activities, pain/discomfort, and anxiety/depression. For each dimension, participants are asked about whether he/she is experiencing any problem and the severity of the problem (no problem, moderate problem, or severe problem). We further categorized the responses as having either no problem or moderate/severe problem. In addition, the EQ-5D index was calculated to reflect the overall health status using the Korean version of the value set, which was developed by the survey of the time trade-off evaluations for the general population in Korea [25]. The EQ-5D index is a continuous value, ranging from −0.171 to 1, with lower values indicating poorer health status and 1 the best. From 2007 to 2012, the EuroQol-visual analogue scale (EQ-VAS) was also measured to assess the global status of their health in the KNHANES. The study participants were asked to select a point on a 20 cm vertical scale to indicate their health status in the range between 0 (worst imaginable health) and 100 (best imaginable health). 

### 2.6. Statistical Analysis

The KNHANES uses a clustered, multistage, stratified, and rolling sampling design. To account for this survey design, all analyses were performed using complex survey analysis. Baseline characteristics of the study population were presented as means with standard errors (SE) and frequencies with weighted proportions. Proportions of having a moderate/severe problem in each dimension of EQ-5D by CKD stages were compared using a Rao-Scott chi-square test. The mean (SE) of EQ-5D index and EQ-VAS across CKD stages were compared using a linear regression model. 

We estimated the odds ratios (ORs) and 95% confidence intervals (CIs) of having problems in each dimension of EQ-5D by CKD stages using multivariable logistic regression analysis. The models were adjusted for age, sex, and BMI (Model 1), further adjusted for residential area, annual income, and education level (Model 2), further adjusted for smoking, alcohol drinking, and physical activity (Model 3), and, finally, further adjusted for the presence of NAFLD and of a chronic disease, including hypertension, stroke, CHD, history of cancer, asthma, COPD, and arthritis (Model 4). We further estimated the ORs of having problems in each dimension of EQ-5D by comparing those with moderate to severe CKD (CKD stages 3–5) to those without (no CKD and CKD stages 1–2). In addition, the EQ-5D index and EQ-5D VAS were compared across CKD stages using multivariable linear regression models with the same model adjustment as above. 

All analyses were performed using Stata 15.0 (StataCorp LLC, College Station, TX, USA).

## 3. Results

Among 7243 participants included in the study, 24.0% (*n* = 1768) had CKD and 8.6% (*n* = 775) had moderate to severe CKD. The mean age (SE) of all participants was 58.3 (0.2) years and 56.9% were male (Table 1). Compared to participants without moderate to severe CKD, those with moderate to severe CKD were more likely to have lower income and lower education. Participants with moderate to severe CKD were also less likely to be current smokers or high-risk alcohol drinkers, and more likely to have comorbidities including hypertension, stroke, CHD, asthma, COPD, arthritis, and a history of cancer.

In participants with DM, the dimension with the highest prevalence of problems was pain/discomfort (30.5%) and the dimension with the lowest prevalence of problems was self-care (6.9%, Table 2). Participants without CKD and those with CKD stage 1–2 had the highest prevalence of problems in the pain/discomfort dimension (30.5% and 24.7%, respectively). Participants with moderate to severe CKD, on the other hand, had the highest prevalence of problems in the mobility dimension. The mean (SE) of the EQ-5D index and EQ-VAS scores were 0.92 (0.002) and 70.5 (0.5) in participants without CKD, respectively. Those with more advanced CKD had a higher prevalence of problems in all dimensions of EQ-5D with a significant increasing trend (Table 2). They also had progressively lower EQ-5D index and EQ-VAS compared to those without CKD. 

In the fully adjusted models, participants with more advanced CKD were more likely to experience problems in each dimension of EQ-5D except the anxiety/depression dimension (Table 3). In particular, compared to those without CKD, the adjusted ORs (95% CI) for having any problem in the usual activities dimension was 1.66 (1.31–2.10) in CKD stage 3 and 4.26 (2.08–8.73) in CKD stage 4–5. Moreover, compared to those without CKD, those with CKD stage 4–5 had significant associations in mobility (OR 5.51, 95% CI 2.64–11.48), self-care (OR 2.04, 95% CI 1.01–4.15), and pain/discomfort dimensions (OR 2.06, 95% CI 1.13–3.77). However, compared to those without CKD, CKD stage 1–2 was not associated with having problems in any of the 5 dimensions. When comparing those with moderate to severe CKD to those without, the adjusted ORs for having a problem were 1.33 (1.08–1.65) in mobility, 1.34 (1.01–1.76) in self-care, 1.87 (1.49–2.36) in usual activities, 1.10 (0.89–1.35) in pain/discomfort, and 1.16 (0.91–1.47) in anxiety/depression dimensions (Table S1).

**Figure 2:**
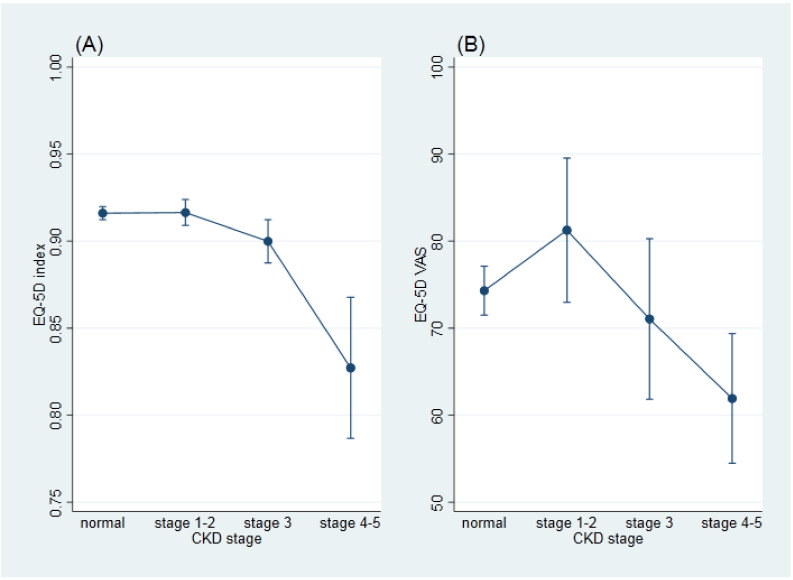
The adjusted EQ-5D index (**A**) and EQ-VAS (**B**) by CKD stage. The adjusted EQ-5D and EQ-VAS were estimated across CKD stage using a linear regression applying complex survey weights. The models were adjusted for age, sex, body mass index, residential area, education level, income, smoking, alcohol drinking, physical activity, and presence of chronic diseases, including hypertension, stroke, coronary heart disease, asthma, chronic obstructive pulmonary disease, arthritis, non-alcoholic fatty liver disease, and a history of cancer.

Participants with stage 3 or stage 4–5 CKD had a significantly lower EQ-5D index than those without CKD in the fully adjusted model (Table S2). In addition, participants with more advanced stages of CKD had both lower EQ-5D index and EQ-VAS (Figure 2). In each dimension of EQ-5D, those reported having a problem in quality of life had significantly lower EQ-VAS (Table S3). The Pearson’s correlation coefficient between EQ-VAS and EQ-5D index was 0.07.

## 4. Discussion

In this nationally representative sample, we investigated the association of CKD on HRQoL in patients with DM. Participants with more advanced stages of CKD were more likely to have problems in most dimensions of EQ-5D after adjusting for socio-demographic and clinical parameters with stronger associations in the mobility and usual activities dimensions. Similarly, participants with CKD stages 3–5 had significantly lower EQ-5D with a progressive degree compared to those without CKD. 

In this study, DM patients with more severe CKD were more likely to have problems in the mobility and usual activities dimensions, which is consistent with previous studies [26,27,28]. In a cross-sectional study of patients with DM and CKD, the mean physical composite summary of the Kidney Disease Quality of Life (KDQOL-36) subscales was lower with more advanced stages of CKD [28]. In addition, in a longitudinal study, those with both DM and CKD had much faster decline in the physical component summary scores measured using SF-36 than those with either disease alone [26]. Although this study used a different metric to measure quality of life, their findings also supported poor HRQoL in mobility and usual activities dimensions among those with both DM and CKD. Moreover, a declining physical component score during the study period was more pronounced in those with more advanced CKD. Similarly, the presence of DM was significantly associated with lower HRQoL, particularly with lower physical function, in CKD patients [29]. Furthermore, in previous studies, poor physical quality of life was associated with a higher risk of mortality in patients with CKD or comorbid DM and CKD [26,30,31]. This suggests that interventions in diabetic patients with early stage CKD to maintain or improve physical function and quality of life may help mitigate declining physical quality of life and mortality.

The association of CKD with having any problem in the anxiety/depression dimension in DM patients was not significant in all stages of CKD. There was also no clear trend in the association between progressive stages of CKD and having any problem in anxiety/depression dimension. Other studies have also found that CKD, even comorbid DM and CKD, was not associated with poorer mental quality of life [26,27,28]. It is possible that the three-level EQ-5D descriptive system may not be sensitive enough to be used as an indicator to determine the anxiety/depression aspect of HRQoL in patients with chronic conditions or with multiple complications. 

We also found that DM patients with CKD stage 1–2 did not have a poor HRQoL in any of the 5 dimensions of EQ-5D or a lower EQ-5D index compared to those without CKD. There are few studies that specifically evaluate how early stage CKD is associated with HRQoL. In another study of CKD patients using the KNHANES data, the EQ-5D index decreased progressively with more advanced stages of CKD [32]. However, there was no difference in the EQ-5D index between CKD stages 1, 2 and no CKD. There are several potential reasons for this finding. In this study, the presence of CKD was defined based on laboratory measurements. The sensitivity of urine dipstick is low in identifying albuminuria [33,34] and, therefore, the prevalence of CKD stages 1 and 2, which is largely determined by the presence of proteinuria, may have been underestimated. Such misclassification would likely result in an underestimation of the association. In addition, EQ-5D may not be very sensitive in detecting any physical or emotional symptoms that present in CKD stages 1 or 2, which are likely to be less debilitating compared to those in more advanced stages of CKD.

There are several strengths to our study. First, this is the largest population-based study of DM patients with CKD on the association of CKD stages with HRQoL. This study showed that more advanced stages of CKD were associated with higher prevalence of problems in each dimension of EQ-5D and lower EQ-5D index even after adjusting for demographic and clinical characteristics. There are few studies about HRQoL in patients with DM and CKD, so our findings provide evidence to support interventions to maintain or improve HRQoL in patients with early stage CKD and DM. Second, EQ-5D is a well-validated and widely used instrument to measure HRQoL. EQ-5D includes 5 different dimensions of HRQoL, which allows us to evaluate the associations of CKD with each aspect of HRQoL separately as well as the overall HRQoL in patients with DM. Indeed, we observed that CKD stages were associated with problems in mobility and usual activities dimensions but not with problems in the anxiety/depression dimension.

Our study has several limitations. First, as a cross-sectional study, we were not able to evaluate the decline in HRQoL with progression of CKD. In addition, as these surveys tend to include individuals who are generally healthier, the prevalence of having problems in each dimension may be underestimated compared to the entire population of DM patients. Second, we used a single measurement of serum creatinine and urine dipstick test to identify CKD and to classify CKD stages. Although the clinical definition of CKD requires at least two measurements measured 6 months apart, it is not feasible in large-scale population-based studies. Furthermore, CKD patients defined with laboratory measurements on a single occasion likely includes those who would not be classified as such using a more strict definition and, thus, our findings may be underestimated. Third, the number of participants with CKD stages 4–5 was small to provide precise estimates, especially when the analysis was performed separately for each dimension of EQ-5D. We, therefore, further estimated the associations of CKD with HRQoL comparing those with moderate to severe CKD (CKD stages 3–5) to those with no CKD or CKD stages 1–2, and also using the EQ-5D index, a composite score of all 5 dimensions of EQ-5D, as the outcome. The results were consistent across these analyses. Fourth, because fasting serum glucose and HbA1c were also measured on a single occasion, we could not confirm the clinical diagnosis of diabetes. Therefore, prediabetes patients might have been included in this study. Finally, our study population was restricted to adults < 80 years since all ages 80 and older were classified as ≥ 80 years in the KNHANES data. The association between CKD stages and HRQoL may be even more relevant in the elderly population and needs to be further explored.

In this study using a national representative sample of Korea, we found that, in DM patients, those with advanced stages of CKD were more likely to have poor HRQoL, particularly in mobility and usual activities, after adjusting for socio-demographic and clinical characteristics. However, we did not observe a clear association between CKD stages and the presence of anxiety/depression, which needs further exploration as the three-level scoring system of EQ-5D may be less sensitive than other tools designed to assess underlying mental health.

## 5. Conclusions

In conclusion, our findings provide evidence that patients with DM have a high prevalence of poor HRQoL and the prevalence increases with more advanced stages of CKD. Therefore, assessment of HRQoL and interventions may be necessary at early stages of CKD and DM.

## Figures and Tables

**Table 1:** Demographic and clinical characteristics of the study participants.

Characteristic	All	without Moderate to Severe Chronic Kidney Disease	with Moderate to Severe Chronic Kidney Disease	*p* Value
No. of participants	7243	6468	775	
No. of participants, weighted	3,361,586	3,071,558	290,028	
Age, y	58.3 ± 0.2	57.4 ± 0.2	68.2 ± 0.4	<0.001
Male, weighted %	3716 (56.9%)	3303 (57.1%)	413 (54.8%)	0.32
BMI, kg/m^2^	25.4 ± 0.1	25.5 ± 0.1	25.3 ± 0.1	0.19
Residence (urban area), weighted %	5427 (78.3%)	4852 (78.3%)	575 (78.0%)	0.84
Income, weighted %				0.71
1st (lowest) quintile	1643 (23.9%)	1452 (23.7%)	191 (26.0%)	
2nd	1479 (20.6%)	1318 (20.7%)	161 (20.3%)	
3rd	1378 (18.4%)	1231 (18.5%)	147 (18.0%)	
4th	1333 (18.0%)	1205 (18.1%)	128 (16.3%)	
5th (highest)	1323 (17.8%)	1189 (17.8%)	134 (17.8%)	
No response	87 (1.3%)	73 (1.3%)	14 (1.7%)	
Education, weighted %				<0.001
≤ Elementary school	3014 (34.7%)	2590 (33.1%)	424 (52.0%)	
Middle school	1169 (15.9%)	1056 (16.0%)	113 (14.6%)	
High school	1885 (29.6%)	1726 (30.3%)	159 (22.1%)	
≥ College	1149 (19.4%)	1077 (20.3%)	72 (9.9%)	
No response	26 (0.3%)	19 (0.3%)	7 (1.4%)	
Smoking status, weighted %				0.04
Never smoker	3851 (48.9%)	3465 (48.8%)	386 (49.5%)	
Ex-smoker	1483 (21.1%)	1299 (20.9%)	194 (25.5%)	
Current smoker	1859 (29.2%)	1665 (29.6%)	184 (23.5%)	
No response	50 (0.8%)	39 (0.7%)	11 (1.5%)	
High-risk alcohol drinking, weighted %	788 (14.3%)	765 (15.4%)	23 (3.2%)	<0.001
Moderate to vigorous physical activity, weighted %	3075 (42.4%)	2804 (43.0%)	271 (34.7%)	<0.001
Hypertension, weighted %	4234 (54.7%)	3654 (52.8%)	577 (75.3%)	<0.001
Stroke, weighted %	421 (5.0%)	312 (4.2%)	109 (13.5%)	<0.001
Coronary heart disease, weighted %	516 (5.9%)	398 (5.2%)	118 (13.2%)	<0.001
History of cancer, weighted %	459 (5.4%)	396 (5.1%)	63 (8.2%)	0.003
Asthma, weighted %	294 (3.6%)	257 (3.5%)	37 (4.6%)	0.20
COPD, weighted %	59 (0.7%)	46 (0.7%)	13 (1.5%)	0.05
Arthritis, weighted %	1591 (18.2%)	1374 (17.3%)	217 (26.9%)	<0.001
Any chronic disease, weighted %	5119 (66.0%)	4445 (64.0%)	674 (87.5%)	<0.001
Nonalcoholic fatty liver disease, weighted %	3708 (52.8%)	3382 (54.2%)	326 (40.5%)	<0.001
HbA1c ≥ 7.0%	3412 (47.3%)	3031 (47.9%)	381 (48.7%)	0.71
CKD, weighted %				
No	5475 (76.0%)	5475 (83.2%)		
1	462 (8.3%)	462 (9.1%)		
2	531 (7.1%)	531 (7.7%)		
3a	557 (6.1%)		557 (70.7%)	
3b	145 (1.6%)		145 (18.6%)	
4	54 (0.7%)		54 (7.9%)	
5	19 (0.2%)		19 (2.8%)	

Values are presented as mean ± standard error (SE) or weighted proportion (%). Abbreviations: BMI, body mass index; CKD, chronic kidney disease; COPD, chronic obstructive pulmonary disease.

**Table 2:** Prevalence of having problems in each dimension of EQ-5D, EQ-5D index, and EQ-VAS by stages of chronic kidney disease.

	All	No CKD	Stage 1–2	Stage 3	Stage 4–5	*p* for Trend
Mobility problem						
*n* (%)	2079 (24.1)	1448 (22.3)	269 (21.7)	311 (41.6)	51 (66.8)	< 0.001
*p*-value *	<0.001					
Self-care problem						
*n* (%)	608 (6.9)	410 (6.2)	71 (5.3)	109 (15.3)	18 (20.4)	< 0.001
*p*-value *	<0.001					
Usual activities problem						
*n* (%)	1278 (14.6)	861 (13.1)	145 (11.5)	235 (31.7)	37 (49.8)	< 0.001
*p*-value *	<0.001					
Pain/discomfort						
*n* (%)	2415 (30.5)	1793 (30.5)	294 (24.7)	291 (39.0)	37 (54.5)	0.01
*p*-value *	<0.001					
Anxiety/depression						
*n* (%)	1104 (13.8)	795 (13.2)	151 (13.9)	146 (19.7)	12 (15.1)	0.003
*c*	<0.001					
EQ-5D index						
Mean ± SE	0.91 ± 0.002	0.92 ± 0.002	0.93 ± 0.004	0.86 ± 0.01	0.79 ± 0.02	<0.001
*p*-value **		Reference	0.05	<0.001	<0.001	
EQ-VAS						
Mean ± SE	70.0 ± 0.4	70.5 ± 0.5	71.5 ± 0.9	63.1 ± 1.4	58.9 ± 2.8	<0.001
*p*-value **		Reference	0.32	<0.001	<0.001	

* *p*-value was estimated for any difference in the prevalence of having problems for each dimension of EQ-5D across stages of chronic kidney disease using Rao-Scott chi-square test. ** *p*-value was estimated for the difference in EQ-5D index and EQ-VAS comparing each stage of chronic kidney disease to no chronic disease using a linear regression model. Abbreviations: CKD, chronic kidney disease; EQ-5D, European Quality of Life Questionnaire Five Dimension; EQ-VAS, EuroQol-visual analogue scale; SE, standard error.

**Table 3:** Odds ratios of having problems in each dimension of EQ-5D by stages of chronic kidney disease.

Variable	Model 1	*p* for Trend	Model 2	*p* for Trend	Model 3	*p* for Trend	Model 4	*p* for Trend
Mobility problem								
No CKD	Reference	<0.001	Reference	<0.001	Reference	<0.001	Reference	0.001
Stage 1–2	1.22 (1.00–1.50)		1.17 (0.95–1.44)		1.15 (0.94–1.42)		1.14 (0.92–1.40)	
Stage 3	1.26 ((1.02–1.56)		1.28 (1.03–1.57)		1.25 (1.01–1.55)		1.14 (0.92–1.42)	
Stage 4–5	6.87 (3.49–13.53)		6.53 (3.18–13.38)		6.31 (3.09–12.88)		5.51 (2.64–11.48)	
Self-care problem								
No CKD	Reference	0.002	Reference	0.003	Reference	0.01	Reference	0.12
Stage 1–2	0.97 (0.70–1.35)		0.93 (0.67–1.30)		0.92 (0.66–1.28)		0.86 (0.62–1.21)	
Stage 3	1.45 (1.10–1.91)		1.45 (1.10–1.91)		1.40 (1.06–1.86)		1.23 (0.92–1.65)	
Stage 4–5	3.07 (1.48–6.36)		2.87 (1.40–5.87)		2.65 (1.30–5.41)		2.04 (1.01–4.15)	
Usual activities problem								
No CKD	Reference	<0.001	Reference	<0.001	Reference	<0.001	Reference	<0.001
Stage 1–2	1.05 (0.83–1.32)		1.00 (0.79–1.27)		0.98 (0.78–1.25)		0.95 (0.74–1.20)	
Stage 3	1.89 (1.52–2.36)		1.90 (1.52–2.37)		1.85 (1.48–2.32)		1.66 (1.31–2.10)	
Stage 4–5	5.91 (3.05–11.46)		5.53 (2.82–10.83)		5.11 (2.61–10.01)		4.26 (2.08–8.73)	
Pain/discomfort								
No CKD	Reference	0.21	Reference	0.36	Reference	0.51	Reference	1.00
Stage 1–2	0.88 (0.74–1.05)		0.85 (0.72–1.02)		0.84 (0.70–1.00)		0.83 (0.70–0.99)	
Stage 3	1.09 (0.89–1.34)		1.07 (0.87–1.32)		1.06 (0.86–1.30)		0.98 (0.78–1.22)	
Stage 4–5	2.46 (1.40–4.35)		2.30 (1.28–4.16)		2.22 (1.22–4.03)		2.06 (1.13–3.77)	
Anxiety/depression								
No CKD	Reference	0.01	Reference	0.02	Reference	0.02	Reference	0.10
Stage 1–2	1.22 (0.97–1.55)		1.19 (0.94–1.50)		1.19 (0.94–1.50)		1.20 (0.94–1.51)	
Stage 3	1.41 (1.10–1.79)		1.36 (1.08–1.73)		1.35 (1.07–1.72)		1.25 (0.98–1.61)	
Stage 4–5	1.08 (0.46–2.56)		0.98 (0.42–2.26)		0.95 (0.41–2.22)		0.80 (0.35–1.82)	

Model 1: adjusted for age, sex and body mass index; Model 2: further adjusted for residential area, education level, and income; Model 3: further adjusted for smoking, alcohol drinking, and physical activity; Model 4: further adjusted for the presence of chronic diseases, including hypertension, stroke, coronary heart disease, asthma, chronic obstructive pulmonary disease, arthritis, nonalcoholic fatty liver disease, and a history of cancer. Abbreviations: CKD, chronic kidney disease; EQ-5D, European Quality of Life Questionnaire Five Dimension; EQ-VAS, EuroQol-visual analogue scale.

## Data Availability

Publicly available datasets were analyzed in this study. This data can be found here: [https://knhanes.kdca.go.kr/knhanes/main.do].

## Supplementary Materials

The following are available online at https://www.mdpi.com/article/10.3390/jcm10204639/s1, Table S1. Odds ratios of having problems in each dimension of EQ-5D comparing those with to those without moderate to severe chronic kidney disease, Table S2. Difference in the EQ-5D index in univariable and multivariable linear regression models, Table S3. Correlation of EQ-VAS with EQ-5D index and the adjusted difference in EQ-VAS for having problems in each dimension of EQ-5D.
