# Supplementary material for: Chronic Kidney Disease on Health-Related Quality of Life in Patients with Diabetes Mellitus: A National Representative Study
Source: J Clin Med. 2021 Oct 10;10(20):4639. doi: 10.3390/jcm10204639 (PMC8540120; doi:10.3390/jcm10204639)
Supplement: Supplementary file 1 [file jcm-10-04639-s001.zip › jcm-1363408-supplementary.pdf]

Table S1. Odds ratios of having problems in each dimension of EQ-5D comparing those with to those without moderate to severe chronic kidney disease.

Dimension	Model 1	Model 2	Model 3	Model 4
Mobility problem	1.47 (1.19–1.81)	1.49 (1.21–1.83)	1.46 (1.18–1.80)	1.33 (1.08–1.65)
Self-care problem	1.58 (1.21–2.07)	1.58 (1.21–2.06)	1.53 (1.17–2.00)	1.34 (1.01–1.76)
Usual activities problem	2.15 (1.73–2.67)	2.16 (1.73–2.68)	2.10 (1.68–2.62)	1.87 (1.48–2.36)
Pain/discomfort	1.22 (1.00–1.49)	1.20 (0.98–1.46)	1.18 (0.97–1.45)	1.09 (0.88–1.34)
Anxiety/depression	1.33 (1.05–1.68)	1.28 (1.02–1.61)	1.27 (1.01–1.60)	1.16 (0.92–1.48)

Model 1: adjusted for age, sex and body mass index; Model 2: further adjusted for residential area, education level, and income; Model 3: further adjusted for smoking, alcohol drinking, and physical activity; Model 4: further adjusted for the presence of chronic diseases, including hypertension, stroke, coronary heart disease, asthma, chronic obstructive pulmonary disease, arthritis, and a history of cancer.

Table S2. Difference in the EQ-5D index in univariable and multivariable linear regression models.

Variable	Univariable	Multivariable
Age, per 1 year increase	−0.003 (−0.004, −0.003)	−0.002 (−0.002, −0.001)
Female, compared to male	−0.058 (−0.065, −0.050)	−0.028 (−0.038, −0.018)
BMI, per 1 kg/m^2 increase	−0.0005 (−0.0016, −0.0005)	−0.001 (−0.002, 0)
Residence area, compared to the urban area	−0.018 (−0.027, −0.009)	−0.006 (−0.014, 0.002)
Income, compared to the lowest quintile		
2	0.028 (0.015, 0.040)	0.023 (0.012, 0.034)
3	0.033 (0.020, 0.045)	0.028 (0.018, 0.039)
4	0.044 (0.031, 0.056)	0.035 (0.024, 0.046)
5 (highest)	0.051 (0.039, 0.063)	0.041 (0.030, 0.051)
Education, compared to the ≤ Elementary school		
Middle school	0.068 (0.057, 0.079)	0.034 (0.023, 0.044)
High school	0.091 (0.081, 0.100)	0.035 (0.025, 0.044)
≥ College	0.115 (0.106, 0.123)	0.039 (0.029, 0.049)
Smoking status, compared to never smoker		
Ex-smoker	0.039 (0.031, 0.048)	−0.003 (−0.014, 0.007)
Current smoker	0.026 (0.018, 0.035)	−0.024 (−0.035, −0.013)
High-risk alcohol drinker	0.036 (0.026, 0.046)	−0.001 (−0.012, 0.009)
Physical activity		
Moderate	0.017 (0.009, 0.025)	0.007 (−0.001, 0.014)
High	0.029 (0.019, 0.039)	0.004 (−0.005, 0.013)
Hypertension	−0.033 (−0.040, 0.026)	−0.001 (−0.008, 0.006)
Stroke	−0.121 (−0.145, −0.096)	−0.090 (−0.113, −0.068)
Coronary heart disease	−0.060 (−0.077, −0.044)	−0.027 (−0.041, −0.012)
History of cancer	−0.006 (−0.021, 0.009)	0.006 (−0.008, 0.019)
Asthma	−0.085 (−0.112, −0.059)	−0.039 (−0.062, −0.016)
COPD	0.012 (0.005, 0.019)	−0.011 (−0.018, −0.004)
Arthritis	−0.120 (−0.131, −0.108)	−0.076 (−0.088, −0.063)
CKD, compared to normal		
Stage 1–2	0.009 (−0.000, 0.019)	0.0002 (−0.008, 0.008)
Stage 3	−0.061 (−0.076, −0.046)	−0.016 (−0.029, −0.003)
Stage 4–5	−0.128 (−0.174, −0.083)	−0.088 (−0.129, −0.048)

The multivariable model is adjusted for age, sex, body mass index, residential area, education level, income, smoking, alcohol drinking, physical activity, and presence of chronic diseases, including hypertension, stroke, coronary heart disease, asthma, chronic obstructive pulmonary disease, arthritis, and a history of cancer. Abbreviations: CKD, chronic kidney disease; COPD, chronic obstructive pulmonary disease.

Table S3. Correlation of EQ-VAS with EQ-5D index and the adjusted difference in EQ-VAS for having problems in each dimension of EQ-5D.

		***p* value**
	Correlation coefficient	
EQ-5D index	0.07	<0.001
Dimension of EQ-5D	Difference in EQ-VAS*	
Mobility problem	−0.218 (−0.231, −0.205)	<0.001
Self-care problem	−0.258 (−0.284, −0.232)	<0.001
Usual activities problem	−0.241 (−0.259, −0.223)	<0.001
Pain/discomfort	−0.179 (−0.191, −0.168)	<0.001
Anxiety/depression	−0.171 (−0.190, −0.151)	<0.001

*The difference in EQ-VAS comparing those with problems to those without in each dimension of EQ-5D was adjusted for age, sex, body mass index, residential area, education level, income, smoking, alcohol drinking, physical activity, and presence of chronic diseases, including hypertension, stroke, coronary heart disease, asthma, chronic obstructive pulmonary disease, arthritis, and a history of cancer. Abbreviations: EQ-5D, European Quality of Life Questionnaire Five Dimension; EQ-VAS, EuroQol-visual analogue scale.
